# Inhibition Profiling of Retroviral Protease Inhibitors Using an HIV-2 Modular System

**DOI:** 10.3390/v7122931

**Published:** 2015-11-27

**Authors:** Mohamed Mahdi, Zsófia Szojka, János András Mótyán, József Tőzsér

**Affiliations:** Laboratory of Retroviral Biochemistry, Department of Biochemistry and Molecular Biology, Faculty of Medicine, University of Debrecen, H-4010 Debrecen, Hungary; atiem3@gmail.com (Z.S.); motyan.janos@med.unideb.hu (J.-A.M.)

**Keywords:** HIV-2, protease, susceptibility, protease inhibitors, modular system

## Abstract

Retroviral protease inhibitors (PIs) are fundamental pillars in the treatment of HIV infection and acquired immunodeficiency syndrome (AIDS). Currently used PIs are designed against HIV-1, and their effect on HIV-2 is understudied. Using a modular HIV-2 protease cassette system, inhibition profiling assays were carried out for protease inhibitors both in enzymatic and cell culture assays. Moreover, the treatment-associated resistance mutations (I54M, L90M) were introduced into the modular system, and comparative inhibition assays were performed to determine their effect on the susceptibility of the protease. Our results indicate that darunavir, saquinavir, indinavir and lopinavir were very effective HIV-2 protease inhibitors, while tipranavir, nelfinavir and amprenavir showed a decreased efficacy. I54M, L90M double mutation resulted in a significant reduction in the susceptibility to most of the inhibitors with the exception of tipranavir. To our knowledge, this modular system constitutes a novel approach in the field of HIV-2 protease characterization and susceptibility testing.

## 1. Introduction

The human immunodeficiency virus type 2 (HIV-2) is a retrovirus that most probably originated from the simian immunodeficiency virus/sooty mangabeys in the Guinea-Bissau region during the 1940s [1]. Since its discovery, the virus had remained relatively contained in the West-African region, with statistical data on its prevalence widely lacking. Current evidence suggests, however, that there is an increasing trend of viral spread beyond its geographical confines, especially in Europe [2,3,4]. HIV-1 and HIV-2 share a similar genomic structure, variability in the genetic sequence accounts for the presence of unique accessory genes; such as viral protein X (vpx) in HIV-2 and viral protein U (vpu) in HIV-1, in addition to sequence and phenotypic differences between envelope proteins [5,6]. Clinically, HIV-2 differs significantly from HIV-1 in that it is much less pathogenic, and HIV-2 infected patients are typically long-term non-progressors to AIDS [7,8]. This feature, undoubtedly, has led to the lack of epidemiological studies on the virus, focusing on HIV-1 as the major pathogen.

Current treatment protocols suggest the use of three nucleoside reverse transcriptase inhibitors (NRTIs) or two NRTIs plus one protease inhibitor (PI) as an initial therapeutic approach against HIV-2 [9,10]. Recently, integrase strand transfer inhibitors have also been shown to exhibit potent efficacy against HIV-2 [11]. Non-nucleoside reverse transcriptase inhibitors (NNRTIs) should not be used in the treatment of HIV-2 infection, as a result of natural polymorphisms in the HIV-2 reverse transcriptase (RT) gene sequence, which alters the binding pocket significantly reducing the binding of the inhibitor, thereby leading to resistance especially against first generation NNRTIs [12]. As protease inhibitors-based regimens constitute a major and effective part in the treatment of HIV infected patients [13,14,15], it is essential to characterize their efficacy against HIV-2.

Ten protease inhibitors were initially approved by the Food and Drug Administration (FDA), out of which nine remain in production today (Table 1). They are typically classified into first and second generation inhibitors, with second generation inhibitors specifically designed to tackle HIV-1 resistance that quickly emerged with first generation drugs, as well as to improve the bioavailability, dosing frequency, and minimize the side effects [16].

**Table 1:** HIV protease inhibitors.

Inhibitor	Abbreviation	Trade Name	Remarks
Saquinavir	SQV	Invirase	
Ritonavir	RTV	Norvir	Used as booster drug
			Combination therapy
Indinavir	INV	Crixivan	
Nelfinavir	NFV	Viracept	
Amprenavir	APV	Agenerase	Discontinued
Lopinavir/+Ritonavir	LPV	Kaletra, Aluvia	Second-generation
			Fixed-dose combination therapy
Atazanavir	ATV	Reyataz	Second-generation
Fosamprenavir	FPV	Telzir, Lexiva	Second-generation
Tipranavir	TPV	Aptivus	Second-generation
			Non-peptidic inhibitor
Darunavir	DRV	Prezista	Second-generation
			Non-peptidic inhibitor

In comparison to HIV-1, few crystal structures of the viral protease (PR) in complex with the inhibitors have been studied for HIV-2 [17]; moreover, the currently approved PIs are essentially designed for HIV-1, and their association with the HIV-2 viral protease had not been thoroughly characterized. To date, a limited number of studies tested the effect of protease inhibitors against HIV-2, either in enzymatic or phenotypic susceptibility assays [18,19,20,21,22,23]. Many factors can influence the results obtained, such as the stability and level of purity of the protease in enzymatic experiments, the type of cells and methods used in cell culture assays, as well as the viral strain. Therefore, the use of a standardized protocol that examines the efficacy of the inhibitor on the same viral enzyme in kinetic and cell culture assays will greatly aid in the determination of a reliable, accurate and comparable IC_50_ values. For this purpose, we have developed an HIV-2 protease modular cassette system [24], which enables the study of the susceptibility of the viral protease to a panel of commercially available PIs, in comparative enzymatic and cell culture experiments.

The viral protease is a homodimeric aspartyl protease, composed of two identical subunits that are 99 amino acids each [25]. The enzyme is crucial for the viral life-cycle by processing the viral Gag and Gag-Pro-Pol viral polyproteins, leading to the formation of mature, infection-competent virions. The literature describes a 39%–48% homology of amino acid sequences between HIV-1 and HIV-2 proteases [26,27], and while the binding site remains highly conserved, this polymorphism alters the specificity of the protease for certain peptide inhibitors, resulting in a decreased binding affinity and, therefore, resistance to treatment.

Treatment-associated mutations in patients receiving PIs are common obstacles in combating AIDS. As a consequence of amino acid substitution(s) in the substrate binding pocket or in a nearby site, the binding affinity of the inhibitors may become substantially reduced, leading to failure in blocking the viral protease [28,29,30]. Of the treatment-associated mutations observed in HIV-2 protease, I54M and L90M have been shown to be implicated in the reduced susceptibility to certain protease inhibitors in phenotypic assays [18,20], but as far as we know, their effects have never been characterized in association with all of the inhibitors, neither enzymatically nor in cell culture. Evidence suggests that resistance to inhibitors may either be a natural phenomenon, as a result of polymorphism in the protease sequence, or may occur selectively under treatment pressure, in sites usually comparable to those found in the HIV-1 protease [18,31,32].

The effects of I54M and L90M single mutations have already been described based on the amprenavir complexes of HIV-1 PR and its drug resistant mutants [33], but to the best of our knowledge, there are no structural data available for I54M and/or L90M mutants of HIV-2 PR. Neither I54 nor L90 HIV-1 PR residues form direct contact with the inhibitor molecule. Substitution of the wild-type amino acid by methionine (Met) introduces a longer side chain, resulting in a shift of the main chain atoms relative to their original positions, in addition to the formation of new van der Waals forces and hydrophobic interactions with the neighboring residues. I54M and L90M single mutations were not found to have a disruptive effect on the secondary or tertiary structure [33].

Ile-54 is positioned in the flap region of the protease, but it is not exposed to the active site cavity. It forms hydrophobic interactions with neighboring flap and loop residues. Substitution of Ile to Met has been found to confer resistance to the protease against certain inhibitors in both HIV-1 and -2 [34,35]. Leu-90 on the other hand, is present in the hydrophobic core of the protease, near the catalytic aspartate residues; but outside of the active site. Substitution of this residue with Met also results in resistance to some inhibitors, again affecting both viruses [18,36,37]. Even though these mutations do not occur in the active site; as already mentioned, it is thought that substitution of the native amino acid to methionine may result in the displacement of certain amino acids, with an overall impact of altering the pocket binding site and the stability of the dimer–inhibitor association, leading to decreased efficacy or resistance to the inhibitor(s) [36,38].

## 2. Results and Discussion

### 2.1. In Vitro Kinetic Assays

In our *in vitro* analysis, following the expression, purification of the protease and confirmation of its activity, kinetic parameters of the enzyme were then determined for both the wild-type and double mutant protease. For the wild-type, *K*_m_ = 0.012 ± 0.002 mM, *k*_cat_ = 0.91 ± 0.02 s^−1^, *k*_cat_/*K*_m_ = 75.8 ± 12.7 mM^−1^·s^−1^, values were published previously [24]. As for the double mutant protease, values were: *K*_m_ = 0.07 ± 0.01 mM, *k*_cat_ = 0.88 ± 0.09 s^−1^, *k*_cat_/*K*_m_ = 12.2 ± 2 mM^−1^·s^−1^. These values suggest that the mutations caused a substantial increase of the *K*_m_ value without affecting the turnover number. Inhibition profiling assays were performed using the high-performance liquid chromatography (HPLC) method in triplicate measurements. Assays using the wild-type HIV-2 protease showed that the majority of the inhibitors (with the exception of nelfinavir, tipranavir and amprenavir) had a good inhibition efficacy against HIV-2 protease (Table 2).

**Table 2:** *In vitro* kinetic inhibition profiling of protease inhibitors for the wild-type and double mutant HIV-2 protease.

Inhibitor	IC_50_ (nM)	*K*_i_ (nM)	IC_50_ (nM)	*K*_i_ (nM)	Fold Increase (*K*_i_)
	Wild-Type		Double Mutant (I54M, L90M)		
Lopinavir	1.18 ± 0.1	0.03 ± 0.001	2.32 ± 0.1	0.32 ± 0.02	10.6
Indinavir	1.30 ± 0.5	0.03 ± 0.02	2.60 ± 1.4	0.36 ± 0.07	12
Darunavir	1.76 ± 0.1	0.05 ± 0.005	8.14 ± 1.3	1.11 ± 0.1	22.2
Saquinavir	3.42 ± 0.1	0.09 ± 0.001	14.09 ± 1.7	1.93 ± 0.2	21.4
Atazanavir	3.34 ± 1	0.09 ± 0.03	10.93 ± 0.2	1.50 ± 0.04	16.6
Ritonavir	5.24 ± 3	0.12 ± 0.075	95.35 ± 17.6	13.05 ± 2.4	108.3
Nelfinavir	38 ± 10	1.01 ± 0.3	190 ± 14.5	26 ± 2	26
Tipranavir	50 ± 2	1.31 ± 0.56	4.80 ± 1.6	0.66 ± 0.2	0.5
Amprenavir	100 ± 7	2.43 ± 1.9	152 ± 9	20.8 ± 1.2	8.5

Data are expressed as mean values ± SD. *K*_i_: Inhibition constant.

Amprenavir, tipranavir and nelfinavir had the highest *K*_i_ values (2.4, 1.3 and 1 nM, respectively); comparing this to a previous study [21], we found that while amprenavir was comparable, tipranavir had a significantly higher *K*_i_. To our knowledge, only one study analyzed the susceptibility of HIV-2 protease kinetically, we speculate that the difference observed in *K*_i_ of some inhibitors could be due to the use of a different HIV-2 strain, a different substrate in the analysis, or perhaps the level of purity and stability of the protease. Indinavir sulfate and lopinavir, on the other hand, had the lowest *K*_i_ (0.03 nM), followed by darunavir and saquinavir (*K*_i_ = 0.05 and 0.09 nM, respectively). Even though atazanavir sulfate had been known to have a variable and somewhat decreased efficacy on different HIV-2 isolates [19,39], to our surprise, its efficacy was comparable to that of saquinavir (*K*_i_ = 0.09 ± 0.03 μM). It is also worth mentioning that no difference was observed between indinavir/atazanavir and their sulfate derivatives (data not shown).

Introduction of the double mutation I54M and L90M greatly decreased the efficacy of the inhibitors, with the exception of tipranavir, which remained indifferent to the mutations. The highest fold increase in *K*_i_ was observed in the case of ritonavir (>100 fold), followed by nelfinavir, darunavir and saquinavir (>20 fold). Indinavir sulfate, atazanavir sulfate and lopinavir showed an increase in *K*_i_ of more than 10 fold, while amprenavir increased by eight fold.

Tipranavir and darunavir are non-peptidic, sulfonamide containing protease inhibitors. In contrast to other protease inhibitors, these molecules are characterized by their flexibility in binding into the active site of the enzyme, giving them an advantage against other peptidomimetic inhibitors should a mutation occur [40,41,42]. Perhaps the reason why we did not observe a significant increase in *K*_i_ in case of the double mutant protease in association with tipranavir can be attributed to nature of the drug’s structure. L90M exerts little or no effect on the susceptibility to tipranavir in HIV-1 isolates, while I54M was associated with a reduced susceptibility to the inhibitor [43]. In regards to darunavir, it is likely that the main chain hydrogen bonds were affected by the mutations, resulting in a decreased binding of the inhibitor as evident by the >20 fold increase in *K*_i_.

### 2.2. Cell Culture Assays

Regarding the cell culture experiments (Table 3), darunavir and lopinavir were very potent inhibitors of the wild-type HIV-2 protease (IC_50_ = 0.42 ± 0.05 and 0.15 ± 0.01 μM, respectively), followed by saquinavir (IC_50_ = 1.3 ± 0.2 μM), indinavir sulfate (IC_50_= 1.4 ± 0.3 μM) and nelfinavir (IC_50_= 2.7 ± 0.9). Tipranavir had an IC_50_ of 3.79 ± 0.6 μM, and the IC_50_ of atazanavir sulfate was 5.9 ± 0.5 μM.

**Table 3:** Inhibition profiling in cell culture using the wild-type and HIV-2 vectors harboring the double mutation.

Inhibitor	IC_50_ (μM)	IC_50_ (μM)	Fold Increase
	Wild-Type	Double Mutant (I54M, L90M)	
Lopinavir	0.1 ± 0.01	3.1 ± 1.1	20.6
Darunavir	0.4 ± 0.05	18.7 ± 1.6	44.5
Saquinavir	1.3 ± 0.2	3.5 ± 1.2	2.7
Indinavir sulfate	1.4 ± 0.3	16.5 ± 2.1	11.7
Nelfinavir	2.7 ± 0.9	5.1 ± 1.7	1.8
Tipranavir	3.7 ± 0.6	5.8 ± 1.7	1.5
Atazanavir sulfate	5.9 ± 0.5	28.6 ± 0.35	4.8
Ritonavir	7.1 ± 0.7	14.5 ± 0.3	2
Amprenavir	68.7 ± 9.2	>100	‒

Data are expressed as mean values ±SD. (-) Fold increase unmeasurable.

The I54M-L90M double mutation resulted in >40 fold and >20 fold increase in IC_50_ in case of darunavir and lopinavir, respectively, and IC_50_ of indinavir sulfate increased by >10 fold. The double mutation resulted in a significant IC_50_ increase of atazanavir sulfate, nelfinavir and saquinavir. As predicted, the double mutation did not have an effect in case of tipranavir.

Despite the dramatic decrease of ritonavir’s inhibition efficacy observed after the introduction of the double mutation in enzymatic experiments, only a two-fold increase of IC_50_ was detected in cell culture assays. Being a potent inhibitor of CYP3A, ritonavir is primarily used as a booster drug for other PI’s that are eliminated by CYP3A metabolism. It had long been successfully used in combination with indinavir and saquinavir, enhancing their bioavailability and potentiating their activity [44].

In contrast to other protease inhibitors, it is important to point out the fact that nelfinavir is metabolized mainly to nelfinavir hydroxy-tert-butylamide (M8) by the P450 enzyme complexes in the liver; particularly CYP2C19. This bioactive metabolite had been shown to exhibit a potent antiviral activity that is comparable to that of the parent drug [45,46]. On a similar note, ritonavir is also biotransformed to M2 by the action of CYP3A4. This active metabolite; however, is found in low concentration in treated human plasma, and had been found to possess a much weaker anti-viral activity as opposed to M8 [47].

### 2.3. Statistical and Correlation Analysis of the Assays

**Figure 1:**
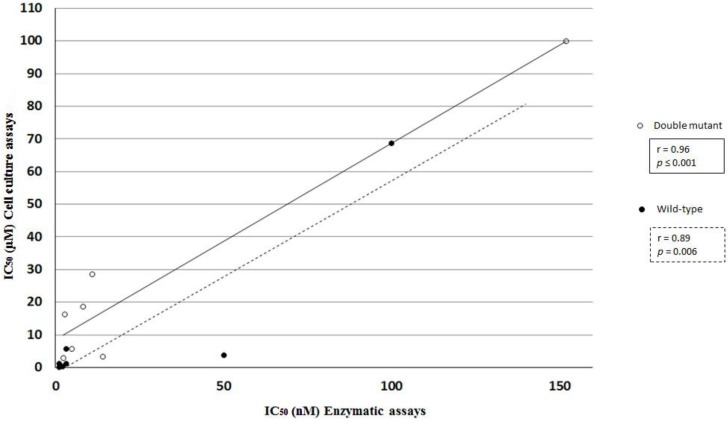
Linear correlation analysis of IC_50_ obtained from *in vitro* enzymatic and cell culture assays using both the wild-type and the double mutant protease. As mentioned previously nelfinavir and ritonavir were excluded from the analysis due to their unique biotransformation properties in cell culture. Correlation in case of the wild-type is indicated by a dotted line, while that of the double mutant is shown by a continuous line. *p* values were calculated at 95% confidence intervals.

Due to their unique pharmacodynamic properties, we have excluded nelfinavir and ritonavir from our correlation analysis, given the fact that HEK 293T cell line was found to express P450 enzyme complexes; albeit in low concentration [48,49]. Their exclusion was necessary to avoid statistical bias in the analysis. Correlating the *in vitro* enzymatic assays to those performed in cell-culture yielded a Pearson’s correlation coefficient of 0.89 (*p* = 0.006) and 0.96 (*p* ≤ 0.001) for the wild-type and the double mutant, respectively (Figure 1).

Further statistical analysis was also performed to complete the linear correlation analysis of data showing non-normal distribution, which revealed that there are no significant differences between the values determined by the different assays (*p* > 0.05) (wild-type: *z* = 1.35 and *p* = 0.22; I54M/L90M mutant: *z* = 0.51 and *p* = 0.69). However, based on the effect size values, the magnitude of the difference was slightly higher in case of the wild-type (effect size value was 0.36 for the wild-type and 0.13 for the double mutant protease).

## 3. Materials and Methods

### 3.1. The Modular System

Our modular system is composed of HIV-2CGP as a structural protein expression construct, CRU5SINCGW; a minimal HIV-2 vector with GFP expression cassette; and pMD.G vector coding for the envelope protein of vesicular stomatitis virus [24]. For the enzymatic assays, pET11a expression plasmid was used to express the viral protease. HIV-2CGP and CRU5SINCGW were a kind gift from Joseph P. Dougherty at the Robert Wood Johnson Medical School (New Brunswick, NJ, USA) [50]. HIV-2CGP was modified to include unique restriction sites (AgeI and AfeI) at 5′ and 3′ of the protease coding region, respectively. These silent mutations were engineered to be 8 amino acids apart from the ends of the protease coding sequence, to allow for the interchange of the protease coding segment between the cell culture CGP vector and the *in vitro* pET11a expression plasmid as described previously [24].

### 3.2. *In Vitro* Protease Expression and Purification

The protease ligated into pET11a was expressed in a culture of *E. coli* BL21 (DE3) cells (Invitrogen, Thermo Fisher Scientific Inc., Waltham, MA, USA). After the disruption of cells by sonication, the protease was then isolated from the inclusion bodies using multiple centrifugation steps in accordance with an HIV protease expression protocol [51]. Thereafter, the protease was purified using reversed-phase high-performance liquid chromatography (RP-HPLC) with the aid of an ÄKTA purifier (Amersham Pharmacia Biotech, Uppsala, Sweden), using a POROS 20 R2 (PE Biosystems, PerSeptive Biosystems, Framingham, MA, USA) C_18_ column [24].

### 3.3. Enzymatic Assays

Following the expression and purification of the protease, its stability and folding were characterized, and the activity was then determined using an oligopeptide substrate representing the protease/reverse transcriptase cleavage site in HIV-2 [24].

Serial dilutions were prepared from the inhibitors using dimethyl sulfoxide (DMSO) in concentrations ranging from 10 nM to 50 μM. The catalytic reactions contained 10 μL buffer E (0.5 M phosphate, 10 mM DTT, 4 M NaCl, 10% glycerol, pH 5.6), 4.8 μL substrate, 5 μL purified protease and 0.2 μL inhibitor in DMSO or DMSO alone (control), followed by incubation at 37 °C for 1 h. The concentration of the protease was adjusted to achieve less than 20% substrate hydrolysis. After incubation at 37 °C for 1 h, the reactions were terminated by the addition of 180 μL 1% trifluoroacetic acid (TFA); thereafter, HPLC measurements were used to determine the inhibitor’s IC_50_ by measuring the decrease in substrate hydrolysis. The inhibitory constant *K*_i_ was then calculated from IC_50_ using the formula *K*_i_ = (IC_50_ − E/2)/(S/*K*_m_ + 1), in which E is the active enzyme concentration, S is the substrate concentration and *K*_m_ is the Michaelis constant.

### 3.4. The Double Mutant Protease

An HIV-2 protease coding sequence identical to the wild-type, yet harboring the I54M and L90M mutations (A162G, C268A) was synthesized and ligated into pET11a expression plasmid utilizing GenScript services (GenScript USA Inc., Piscataway, NJ, USA). The protease sequence was verified by sequencing and restriction analysis. AgeI and AfeI endonucleases were then used to restrict the sequence for subsequent ligation into the CGP vector for cell culture experiments.

### 3.5. Cell Culture Assays

In accordance with an HIV-1 transfection protocol [52], 293T human embryonic kidney cells (Invitrogen) were seeded in T75 flask in 15 mL Dulbecco’s modified Eagle’s medium (DMEM) (Sigma-Aldrich, St. Louis, MO, USA) supplemented with 10% fetal bovine serum (FBS), 1% glutamine and 1% penicillin-streptomycin. The day before the transfection, cells were passaged in order to achieve 70% confluency the next day. At 70% confluency (5–6 × 10^6^ cells/mL), a total of 45 μg plasmid DNA was used for the transfection of cells using *Polyethylenimine*. Cells were then incubated at 37 °C, 5% CO_2_ in 5 mL 1% FBS containing DMEM without antibiotics. After 5–6 h, cells were split and transferred into a 96-well plate containing serial dilutions of the inhibitor ranging from 3.2 nM to 100 µM in a total volume of 200 μL DMEM/well, supplemented with 10% FBS, 1% glutamine and 1% penicillin-streptomycin. After 3 days incubation at 37 °C, the virus-containing medium was collected from the wells, briefly centrifuged to remove cellular debris, and 10 μL samples were taken from each corresponding well. Reverse transcriptase colorimetric assay (catalog No. 11468120910; Roche Applied Science, Mannheim, Germany) was then used to determine the IC_50_ values from triplicate measurements. This ELISA based method quantitatively determines RT activity in cell culture. In order to get accurate results using the colorimetric assay, a slight modification to the manufacturer’s protocol was needed; the incubation of samples with the reaction mixture was carried out for 17–18 h to allow for sufficient detection and quantification of reverse transcriptase.

### 3.6. Protease Inhibitors

The protease inhibitors darunavir, saquinavir, lopinavir, tipranavir, indinavir sulfate and atazanavir sulfate were obtained through the NIH AIDS Reagent Program, Division of AIDS, NIAID, NIH (Germantown, MD, USA). Ritonavir was obtained from Abbott laboratories (Irving, TX, USA), nelfinavir from Agouron, indinavir from MERCK & CO (Keilworth, NJ, USA), atazanavir from *Bristol*-Myers Squibb (New York, NY, USA), and amprenavir from Vertex Pharmaceuticals Inc (Boston, MA, USA).

### 3.7. Statistical Analysis

Normal distribution of the data was tested with the Shapiro–Wilk test. Differences of the IC_50_ values obtained from *in vitro* enzymatic and cell culture assays were calculated for both enzymes. The datasets did not follow the normal distribution; thus, the non-parametric Wilcoxon-test was applied. The null hypothesis (H_0_) was that IC_50_ values obtained had the same mean rank, and the alternative hypothesis (H_1_) was that the mean ranks of the IC_50_ values differed according to the determination method used, using a type I error of 0.05. Due to the small sample size, the Monte-Carlo permutation (based on 99,999 random assignments) were applied to control the asymptotic probability value of the tests [53,54]. Effect size quantifies the size of the difference between two groups of data (*i.e.*, data obtained by enzymatic and cell culture assays) in a standardized and comparable form. It ranges from −1 to +1, where 0 means that the methods have no effect on the IC_50_ values; values approaching −1 or +1 indicate larger magnitude [55,56]. Statistical tests were performed with PAST 3.09 software [57].

## 4. Conclusions

In conclusion, we believe this to be the first study to analyze the susceptibility of HIV-2 to a complete panel of protease inhibitors using combined *in vitro* enzymatic and cell culture-based inhibition profiling. Analyzing results obtained from both assays, we can safely conclude that lopinavir, darunavir, indinavir sulfate and saquinavir are very potent HIV-2 protease inhibitors, as evident from the low *K*_i_ and IC_50_ values calculated. Tipranavir, nelfinavir and atazanavir sulfate, on the other hand, showed a significantly lower efficacy when compared to the others. Furthermore, amprenavir failed to potently inhibit HIV-2 protease, especially in cell culture experiments.

A major limitation of studies on protease inhibitors is the variability in results obtained. The values obtained from our cell culture assays were significantly higher than those obtained from other studies on HIV-2 [18,19,20]; as mentioned earlier, the type of assay used and cell culture as well as the viral strain under examination can greatly influence the results. This variability in results serve as hindrance to the analysis in determining the efficacy of protease inhibitors in regards to HIV-2; therefore, our aim was to assay for all of the currently marketed protease inhibitors using the same HIV-2 protease coding region in comparative *in vitro* kinetic and cell culture assays. We hope that this methodology, as well as the results obtained from our experiments may help tailor the use of protease inhibitors in HIV-2 infected patients.
